# Cardiovascular risk factors and frailty in a cross-sectional study of older people: implications for prevention

**DOI:** 10.1093/ageing/afy080

**Published:** 2018-05-22

**Authors:** Tsz Yan Wong, M Sofia Massa, Aisling M O'Halloran, Rose Ann Kenny, Robert Clarke

**Affiliations:** 1 Nuffield Department of Population Health, University of Oxford, Oxford, UK; 2 TILDA, Department of Medical Gerontology, Trinity College, Dublin, Ireland

**Keywords:** cardiovascular risk factors, frailty, prevention, older people

## Abstract

**Objective:**

to examine the associations of cardiovascular disease (CVD) and cardiovascular risk factors with frailty.

**Design:**

a cross-sectional study.

**Setting:**

the Irish Longitudinal Study on Ageing (TILDA).

**Participants:**

frailty measures were obtained on 5,618 participants and a subset of 4,330 participants with no prior history of CVD.

**Exposures for observational study:**

cardiovascular risk factors were combined in three composite CVD risk scores (Systematic Coronary Risk Evaluation [SCORE], Ideal Cardiovascular Health [ICH] and Cardiovascular Health Metrics [CHM]).

**Main outcome measures:**

a frailty index (40-items) was used to screen for frailty.

**Methods:**

the associations of CVD risk factors with frailty were examined using logistic regression.

**Results:**

overall, 16.4% of participants had frailty (7.6% at 50–59 years to 42.5% at 80+ years), and the prevalence was higher in those with versus those without prior CVD (43.0% vs. 10.7%). Among those without prior CVD, mean levels of CVD risk factors were closely correlated with higher frailty index scores. Combined CVD risk factors, assessed using SCORE, were linearly and positively associated with frailty. Compared to low-to-moderate SCOREs, the odds ratio (OR) (95% confidence interval, CI) of frailty for those with very high risk was 3.18 (2.38–4.25). Conversely, ICH was linearly and inversely associated with frailty, with an OR for optimal health of 0.29 (0.21–0.40) compared with inadequate health.

**Conclusions:**

the concordant positive associations of SCORE and inverse associations of ICH and CHM with frailty highlight the potential importance of optimum levels of CVD risk factors for prevention of disability in frail older people.

## Introduction

Frailty is a multi-dimensional condition that is common in older people, characterised by decreased physiological reserve and associated increased risk of falls, hospitalisation, nursing home admission and death. The prevalence of frailty in community-dwelling individuals aged 65 years or older varies between 4% and 59% and increases with age [1]. Screening to detect individuals with frailty is important as it is associated with modifiable risk factors for disability and death. Several studies have reported associations of cardiovascular disease (CVD) risk factors with frailty, but few studies have examined the effects of composite CVD risk scores for prediction of frailty [2–5].

The European Society of Cardiology’s Systematic Coronary Risk Evaluation (SCORE) [6] estimates the 10-year absolute risk of CVD death in relation to age, sex, smoking, total cholesterol and systolic blood pressure (SBP). Moreover, the American Heart Association’s Ideal Cardiovascular Health (ICH) score and Cardiovascular Health Metrics (CHM) [7] estimate risk of cardiovascular health among people with no prior history of CVD. While there is no consensus on the optimum instrument to assess frailty [8–10], the frailty index measures multi-dimensional deficits in individuals and is believed to be better than other frailty measures as a predictor for adverse outcomes [11, 12]. The aims of the present study are: (i) to compare the prevalence of frailty in community-dwelling individuals aged 50 years or older with and without a prior history of CVD and (ii) to examine the associations of several composite CVD risk scores (SCORE, ICH and CHM) with frailty in a subset of participants with no prior history of CVD.

## Methods

### Participants

The present analysis used data from the baseline survey of the Irish Longitudinal Study on Ageing (TILDA), which recruited 8,175 participants aged 50 years or older, and an additional 329 spouses aged <50 years in 2009–11 [13]. After providing informed consent, participants completed a computer-aided personal interview at home [13] and clinical measurements were collected at a health centre or at home [14, 15]. For the present report, 2,350 individuals who did not attend the health assessment, 266 individuals aged <50 years or who had missing data on age and 270 individuals with missing data on outcomes were excluded (see Supplementary Figure S1 available at *Age and Ageing* online).

Frailty index was calculated in 5,618 participants for comparisons of the associations of frailty in those with versus those without a prior history of CVD. In order to exclude diseases that may cause frailty phenotypes as a result of a single disease, the associations with CVD risk factors were restricted to a subset of 4,330 individuals without prior CVD, medication use for depression, cognitive impairment or Parkinson’s disease [8]. Ethics approval was granted by the Trinity College Research Ethics Committee and all participants provided written informed consent [14, 15].

### Frailty outcomes

Frailty was detected using the frailty index (see Supplementary Table S1, available at *Age and Ageing* online) calculated based on 40 self-reported variables involving multiple domains, representing different dimensions of health in older people [16, 17]. The included dichotomous measures were coded as 0 and 1 (i.e. 0 for absence, and 1 for presence of deficits). The ordered categorical measures were coded as a fraction proportional to the number of responses (e.g. five categories [0, 0.25, 0.5, 0.75, 1.0] ranging from none to all deficits). The participant’s frailty index score was calculated by dividing the number of deficits recorded by the total number of measures. Consistent with previous studies, individuals with a frailty index score >0.25 were defined as having frailty [18].

### Cardiovascular risk factors

Estimation of SCORE involved age (years), sex, current smoking status (yes/no), total cholesterol (mmol/l) and SBP (mmHg) (see Supplementary Table S2, available at *Age and Ageing* online). Among the 4,330 individuals, 28.4% and 21.8% reported use of blood pressure-lowering or cholesterol-lowering medication, respectively. In order to account for treatment effects, values of SBP and DBP were increased by 10 mmHg and 5 mmHg, respectively, for any individuals who reported current use of blood pressure-lowering medication [19]. Likewise, values of total cholesterol were increased by 1 mmol/l for individuals who reported current use of cholesterol-lowering medication [20]. Individuals were categorised into low-to-moderate risk (SCORE < 5%), high risk (5% ≤ SCORE < 10%) and very high risk (SCORE ≥ 10%) [21] absolute risks of death from CVD in the next 10 years.

ICH data were available for six domains: (i) never-smokers and past smokers who quit ≥2 years, (ii) body mass index (BMI) <25 kg/m^2^, (iii) ideal physical activity, (iv) untreated total cholesterol <5.2 mmol/l, (v) untreated SBP <120 mmHg and diastolic blood pressure (DBP) <80 mmHg and (vi) absence of diabetes, but no data were available on healthy diet (see Supplementary Table S2, available at *Age and Ageing* online). For CHM, scores of 0, 1 and 2 were allocated to those with poor, intermediate and ideal metrics, respectively (see Supplementary Table S3, available at *Age and Ageing* online) [3]. ICH (maximum score 6) was classified as inadequate (0–2), average (3) and optimal health (4–6). Likewise, CHM (maximum score 12) was classified as inadequate (0–5), average (6–7) and optimal health (8–12).

### Statistical analyses

Values with missing data were substituted using age- and sex-specific mean, median or mode values in the small number of individuals with missing data (5.8% of 5,618 participants). Potential confounders were: age, sex, education, household wealth, cognitive function and depression. The association of prior CVD with frailty was assessed in all participants using chi-square tests. The association of CVD risk factors with frailty was assessed in the subset with no prior history of CVD. The log of frailty index was regressed against age. Thirty items defining the frailty index were randomly selected to examine if such relationships were sensitive to any missing deficits and this procedure was repeated 10 times [17].

Frailty was regressed against individual CVD risk factors of SCORE and ICH/CHM, separately, after adjustment for all relevant confounders. For the main analysis, unadjusted models for SCORE and the age-adjusted models for ICH/CHM were initially conducted. Subsequent analyses for all risk scores were sequentially adjusted for sex (only for ICH/CHM models), education, household wealth, cognitive function and depression. The odds ratios (ORs) of frailty and 95% confidence intervals (95% CI) were presented for incremental (SCORE) or decreasing (ICH/CHM) levels of CVD risk scores. The 95%CI are presented both as conventional CI in the text and on a floating absolute scale in the Figure. Likelihood ratio tests were used to assess the presence of any significant trends. Sensitivity analyses for the main models were conducted using blood pressure and total cholesterol without correction for blood pressure-lowering medication or cholesterol lowering medication, respectively. Additional sensitivity analyses were performed with frailty defined as a frailty index of ≥0.20 [22] and ≥0.21 [23], respectively. Further sensitivity analyses were conducted after excluding high blood pressure and high cholesterol as two of the deficits in the frailty index (to avoid reverse causality bias), leaving 38 deficits in the revised frailty index for analyses with composite CVD risk scores. All *P*-values were reported as two-sided. All statistical analyses were performed using STATA 14.0 (StataCorp, College Station, TX, USA).

### Participant involvement

Participants were informed about the design and methodology of the TILDA study.

## Results

### Characteristics of participants with and without a history of prior cardiovascular disease

Overall, 16.4% of the 5,618 participants were defined as having frailty and the prevalence increased with age (7.6% at age 50–59 years to 42.5% at age 80+ years) and was higher in those with versus those without prior CVD (43.0% vs. 10.7%) (*P* < 0.001) (Table 1). Overall, the 5,618 participants had a median (Interquartile range [IQR]) age of 62 (55–69) years and 46.1% were males.

**Table 1.** Age-specific prevalence of frailty, by presence or absence of prior CVD

Age (years)	No prior CVD (*n* = 4,624)	Prior CVD (*n* = 994)	All (*n* = 5,618)
50–59	127 (5.9%)	52 (24.3%)	179 (7.6%)
60–69	171 (10.9%)	131 (41.1%)	302 (15.9%)
70–79	135 (18.8%)	173 (50.9%)	308 (29.1%)
≥80	60 (32.1%)	71 (58.7%)	131 (42.5%)
All	493 (10.7%)	427 (43.0%)	920 (16.4%)

Values presented are *N* (%).

### Baseline characteristics

The 4,330 participants without prior CVD had a median IQR age of 60 (55–67) years and 44.5% were males (see Supplementary Table S4, available at *Age and Ageing* online). The median (IQR) cardiovascular risk SCORE was 2.4% (1.1–5.4) per 10 years. Also, 72.8%, 16.4%, and 10.8% of the individuals had low-to-moderate, high and very high 10-year risks of fatal CVD, respectively. Overall, 94.3% did not have diabetes, 82.9% were never-smokers or had quit for 2 years or more, 45.6% had ideal physical activity, 42.2% had ideal blood cholesterol, 23.5% had ideal body mass and 16.8% had ideal blood pressure. After categorisation of ICH, 30.2%, 38.2% and 31.6% of the participants had inadequate, average and optimal cardiovascular health. Likewise for CHM, 14.5%, 35.1% and 50.4% participants had inadequate, average and optimal cardiovascular health.

### Distribution of frailty index

The median (IQR) frailty index score in 4,330 participants was 0.10 (0.06–0.17) and 99th percentile and maximum values were 0.40 and 0.58, respectively. The apparent linear association in the quantile–quantile plot demonstrated that the frailty index had a Gamma distribution (see Supplementary Figure S2, available at *Age and Ageing* online). Regression of the frailty index by age indicated an exponential accumulation of frailty deficits of 3% per year. The distributions of components of the frailty index in all participants and subset without prior CVD are shown in Supplementary Figure S3a and S3b, available at *Age and Ageing* online.

### Associations of cardiovascular risk factors with frailty

For components of CHM, higher levels of BMI were linearly and positively associated with risks of frailty. In contrast, physical activity was linearly and inversely associated with risk of frailty. Compared to individuals with no diabetes and non-current smokers, having diabetes or being a current smoker was also positively associated with frailty. Compared to their respective baseline groups of SCORE components, higher age, female sex and current smoking were also positively associated with frailty. Blood pressure-lowering medication and cholesterol-lowering medication were used by one-half and one-third, respectively, of those in the top quintile of the frailty index (Table 2). For blood pressure and total cholesterol levels, only SBP as a SCORE component was positively associated with frailty.

**Table 2.** Distribution of cardiovascular risk factors, by quintiles of frailty index in 4,330 participants with no prior history of CVD

% or mean (SD)	Quintiles of frailty index				
	I	II	III	IV	V
Range of frailty index	(0.00–0.05)	(0.05–0.08)	(0.09–0.13)	(0.13–0.19)	(0.19–0.58)
Demography/medical history					
Age, years	57.6 (6.3)	59.7 (7.3)	61.6 (7.9)	63.3 (8.5)	66.2 (8.9)
Sex, female	47.9	50.7	51.8	60.6	67.2
Current smokers	14.9	14.8	15.6	16.0	16.6
Diabetes	0.9	1.6	3.4	8.9	14.1
BP-lowering medication	5.9	19.7	26.1	37.0	54.9
Cholesterol-lowering medication	5.1	14.8	20.7	30.7	38.6
Clinical measurements					
SBP, mmHg	132.5 (18.8)	137.0 (21.6)	139.3 (20.9)	140.1 (20.4)	143.1 (21.3)
DBP, mmHg	83.0 (11.1)	84.6 (11.9)	84.7 (11.5)	84.8 (10.9)	85.0 (11.5)
BMI, kg/m^2^	27.4 (4.0)	27.9 (4.5)	28.4 (4.5)	29.0 (5.4)	30.0 (5.6)
Total cholesterol, mmol/l	5.5 (1.0)	5.5 (1.0)	5.5 (1.0)	5.5 (1.0)	5.4 (1.0)

BMI, body mass index; BP, blood pressure; DBP, diastolic blood pressure; SBP, systolic blood pressure; SD, standard deviation.

### Associations of composite cardiovascular risk scores with frailty

**Figure 1:**
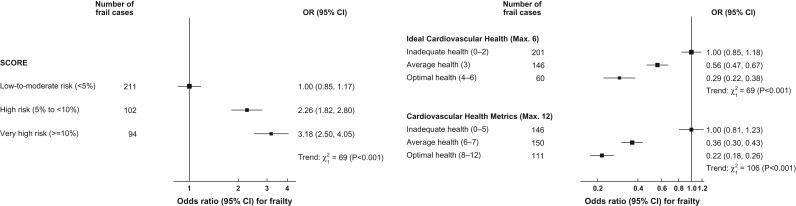
Association of systematic coronary risk evaluation score (left) and of ideal cardiovascular health and cardiovascular health metrics with frailty (right). Odds ratios (OR) are presented on a floating absolute scale. Each square has area inversely proportional to the variance of the log OR. The horizontal lines indicate 95% confidence intervals. The vertical line corresponds to an OR of 1.0. The analyses were adjusted for sex, age, education, household wealth, cognitive function and depression, where appropriate.

Compared to low-to-moderate risk, the unadjusted ORs (95% CI) of frailty for high risk and very high-risk categories of SCORE were 2.34 (1.82–3.01) and 3.51 (2.69–4.58), respectively. Compared to inadequate health in ICH, the age-adjusted ORs of frailty for average and optimal cardiovascular health were 0.51 (0.41–0.65) and 0.25 (0.19–0.34). Likewise, compared to inadequate health in CHM, the age-adjusted ORs of frailty for average and optimal health were 0.34 (0.26–0.44) and 0.18 (0.14–0.24). SCORE was linearly positively associated with frailty (*P*_trend_ < 0.001). In addition, there were linear inverse associations of ICH and CHM with frailty (*P*_trend_ < 0.001). Compared to low-to-moderate risk, the adjusted ORs of frailty were 2.26 (1.73–2.95), and 3.18 (2.38–4.25) for high and very high risk of SCORE (Figure 1). For ICH, the adjusted ORs of frailty for average and optimal cardiovascular health were 0.56 (0.44–0.71) and 0.29 (0.21–0.40) compared with those with inadequate cardiovascular health (Figure 1). For CHM, the adjusted ORs of frailty were 0.36 (0.28–0.48) and 0.22 (0.16–0.29) for average and optimal health compared to inadequate health, respectively (Figure 1). The results did not differ materially when SCORE and ICH/CHM were not corrected for the use of medications for blood pressure and blood cholesterol (see Supplementary Figures S4 and S5, available at *Age and Ageing* online) or when the cut-point for frailty was changed (Data not shown). The analyses were also unaltered after removing hypertension and high cholesterol from the frailty index (and using 38-item instead of 40-item frailty index: Data not shown).

## Discussion

Almost 1 in 6 of the study population had evidence of frailty, but the prevalence increased with age and was also 4-fold greater in those with versus those without prior CVD (43% vs. 11%). Among individuals who were free of CVD, the combined effects of classical CVD risk factors using European coronary risk SCORE were linearly and positively associated with risk of frailty. Likewise, the American Heart Association metrics of ICH and CHM, were both linearly inversely associated with risk of frailty, independent of age and sex. Analysis of quintiles of frailty index showed a greater burden of CVD risk factors in individuals with higher levels of frailty.

We adjusted the analyses for potential confounders to be consistent with those adopted in previous studies [2, 3]. The results of the present study are also consistent with previous epidemiological evidence indicating positive associations of smoking [24], diabetes [25] and obesity [4] with frailty or disability, and randomised trial evidence on the protective effects of physical activity to prevent the complications of frailty [26]. One of the limitations of the present study was the cross-sectional design and, hence, it was unable to infer causality of cardiovascular risk factors for frailty, but the strong correlation of composite CVD risk scores with frailty highlights their potential importance for prevention of disability in older people.

The results of observational studies indicate weaker associations of blood pressure and cholesterol with CVD in older versus middle aged individuals, but randomised trials demonstrate similar proportional effects of lowering total cholesterol or blood pressure at all ages [20, 27, 28]. Analyses of the SPRINT trial and the HYpertension in the Very Elderly Trial demonstrated comparable proportional reductions in risk of major vascular events in individuals with different frailty statuses [27, 28].

While levels of blood pressure and cholesterol were corrected for medication use to minimise reverse causation, cross-sectional analysis could not fully exclude the possibility of reverse causation. Likewise, the estimates for prevalence of frailty may possibly underestimate those in the Irish or UK population as the data were not weighted for the age structure of such populations. The prevalence may also have been underestimated due to healthy volunteer effect, as only individuals who attended the health assessment were included. The results generated from this national-representative cohort were generalisable to community-dwelling individuals aged 50 years or older who were free of prior CVD in Ireland. In addition, SCORE is only valid for individuals aged less than or equal to 65 years, and older people would have high CVD risk due to their advancing age [21]. The absolute values for SCORE may have been inflated as the age-standardised mortality rate of all vascular diseases declined by 34.9% during 2003–12 in Ireland, but it should not affect their ability to rank individuals [29].

The concordant positive association of SCORE with frailty, and of the inverse associations of ICH and CHM with frailty, reinforce the importance of CVD risk factors for frailty. Indeed, the revised contract for General Practitioners in the UK for 2017–18 includes advice to screen older people for frailty using the electronic frailty questionnaire [30] and advocates strategies to review medication of frail older people each year. Hence, screening older people to identify frailty could include opportunities to review lifestyle advice and medication to optimise levels of CVD risk factors for prevention of disability and death in frail older people. More evidence is needed about the effects of lowering blood pressure or cholesterol in older people at varying severity of frailty before recommending drug treatments in all such high-risk older people.

**Key points**

- Overall, 16% of the population had frailty (8% at 50-59 and 43% at 80+ years).
- The prevalence of frailty was higher in those with versus those without prior CVD (43% vs 11%).
- Among those without prior CVD, mean levels of CVD risk factors were closely correlated with frailty index scores.
- The concordant positive associations of SCORE and ICH with frailty reinforce the importance of CVD risk factors for frailty.
- Screening older people for frailty should be accompanied by consideration for cardiovascular risk factor modification.

## Supplementary Material

Supplementary Data

## Transparency

All authors state that the manuscript is an honest, accurate, and transparent account of the study being reported; that no important aspects of the study have been omitted; and that any discrepancies from the study as planned have been explained.

## Data sharing

Anonymised participant level data of The Irish Longitudinal Study on Ageing (TILDA) Wave 1, 2009–11 were accessed via the Irish Social Science Data Archive—www.ucd.ie/issda. Data sharing: all data are accessible from the Irish Social Science Data Archive.
