# Supplementary material for: Cardiovascular risk factors and frailty in a cross-sectional study of older people: implications for prevention
Source: Age Ageing. 2018 May 22;47(5):714–20. doi: 10.1093/ageing/afy080 (PMC6108388; doi:10.1093/ageing/afy080)
Supplement: Supplementary Data [file aa-17-1113-file003.docx]

**Supplementary Material**

**Cardiovascular risk factors and frailty in a cross-sectional study of older people: implications for prevention**

|  | |  | Page |
| --- | --- | --- | --- |
|  | |  |  |
| Table of Contents | | | 1 |
|  |  | |  |
| Supplement Table 1: | Components of the 40-item frailty index | | 2 |
|  |  | |  |
| Supplement Table 2: | Components of SCORE and Ideal Cardiovascular Health/Cardiovascular Health Metrics (ICH/CHM) in TILDA | | 3 |
|  |  | |  |
| Supplement Table 3: | Categories of Ideal Cardiovascular Health and Cardiovascular Health Metrics | | 4 |
|  |  | |  |
| Supplement Table 4: | Selected characteristics of all 4330 participants and the subset with frailty | | 5 |
|  |  | |  |
| Supplement Figure1: | CONSORT diagram of all participants and subset with no prior history of CVD | | 6 |
|  |  | |  |
| Supplement Figure 2: | Quantile-quantile plot of the frailty index in 4330 participants versus Gamma distribution with scale parameter ***λ*** (16.26) and shape parameter *k* (2.02) | | 7 |
|  |  | |  |
| Supplement Figure 3a: | Frequency distribution of 40-item components of the frailty index  in 4330 participants with no prior history of CVD | | 8 |
|  |  | |  |
| Supplement Figure 3b: | Frequency distribution of 40-item components of the frailty index  in all 5618 participants | | 8 |
| Supplement Figure 4: | Association of systemic coronary risk evaluation with frailty, without correction for use of medication | | 9 |
|  |  | |  |
| Supplement Figure 5: | Associations of Ideal Cardiovascular Health (ICH) and Cardiovascular Health Metrics (CHM) with frailty, without correction for medication | | 10 |

**Supplement Table 1. Components of the 40-item frailty index**

| **Variables in TILDA** | **Cut-points** |
| --- | --- |
| 1. Difficulty walking 100 m | Yes=1; No=0 |
| 2. Difficulty jogging 1.5 km | Yes=1; No=0 |
| 3. Difficulty rising from chair | Yes=1; No=0 |
| 4. Difficulty climbing several flights of stairs | Yes=1; No=0 |
| 5. Difficulty climbing one flight of stairs | Yes=1; No=0 |
| 6. Difficulty stooping, kneeling, or crouching | Yes=1; No=0 |
| 7. Difficulty reaching above shoulder height | Yes=1; No=0 |
| 8. Difficulty pushing/pulling large objects | Yes=1; No=0 |
| 9. Difficulty lifting/carrying weights ≥10 lb | Yes=1; No=0 |
| 10. Difficulty picking up coin from table | Yes=1; No=0 |
| 11. Difficulty preparing a hot meal | Yes=1; No=0 |
| 12. Difficulty with household chores | Yes=1; No=0 |
| 13. Difficulty shopping for groceries | Yes=1; No=0 |
| 14. Feeling lonely | Rarely or none of the time=0;  Some or a little of the time=0.33;  Occasionally or a moderate amount of time=0.66;  All of the time (5-7 days)=1 |
| 15. Poor self-rated physical health | Excellent=0; Very good=0.25; Good=0.5;  Fair=0.75; Poor=1 |
| 16. Poor self-rated vision | Excellent=0; Very good=0.25; Good=0.5;  Fair=0.75; Poor=1 |
| 17. Poor self-rated hearing | Excellent=0; Very good=0.25; Good=0.5;  Fair=0.75; Poor=1 |
| 18. Difficulty following a conversation with one person | None=0; Some=0.5; Much/Impossible=1 |
| 19. Daytime sleepiness | Would never doze=0;  Slight chance of dozing=0.33;  Moderate chance of dozing=0.66;  High chance of dozing=1 |
| 20. Polypharmacy (≥5 medications) | Yes=1; No=0 |
| 21. Intrusive pain | Yes=1; No=0 |
| 22. Knee pain | Yes=1; No=0 |
| 23. Urinary incontinence | Yes=1; No=0 |
| 24. Hypertension or high blood pressure | Yes=1; No=0 |
| 25. Angina | Yes=1; No=0 |
| 26. Heart attack | Yes=1; No=0 |
| 27. Diabetes | Yes=1; No=0 |
| 28. Stroke | Yes=1; No=0 |
| 29. Transient ischemic attack | Yes=1; No=0 |
| 30. High cholesterol | Yes=1; No=0 |
| 31. Irregular heart rhythm | Yes=1; No=0 |
| 32. Other cardiovascular disease | Yes=1; No=0 |
| 33. Cataracts | Yes=1; No=0 |
| 34. Glaucoma | Yes=1; No=0 |
| 35. Age related macular degeneration | Yes=1; No=0 |
| 36. Chronic lung disease | Yes=1; No=0 |
| 37. Arthritis | Yes=1; No=0 |
| 38. Osteoporosis | Yes=1; No=0 |
| 39. Cancer | Yes=1; No=0 |
| 40. Varicose ulcer | Yes=1; No=0 |

Adapted and modified from O'Connell et al. [16]

Details of questionnaires and derived variables are available at <http://www.ucd.ie/issda/static/documentation/tilda/tilda-capi-qaire-wave1.pdf> and <https://www.ucd.ie/t4cms/Derived%20Variables%20Codebook_%20wave%201%20v1.6.docx>.

**Supplement Table 2. Components of SCORE and Ideal Cardiovascular Health/Cardiovascular Health Metrics (ICH/CHM) in TILDA**

| **Components** | **SCORE^1^** | **Ideal Cardiovascular Health/**  **Cardiovascular Health Metrics^2^** |
| --- | --- | --- |
|  |  |  |
| Age | + |  |
| Sex | + |  |
| Smoking | + | + |
| Total cholesterol | + | + |
| Systolic blood pressure | + | + |
| Diastolic blood pressure |  | + |
| Body mass index |  | + |
| Blood glucose |  | Changed to diabetes |
| Physical activity |  | + |
| Healthy diet |  | Not available in TILDA |
|  |  |  |

^1^ Conroy et al. [6]

^2^ Lloyd-Jones et al. [7]

**Supplement Table 3. Categories for Ideal Cardiovascular Health and Cardiovascular Health Metrics**

|  | **Equivalent variables in TILDA** | | |
| --- | --- | --- | --- |
|  | **Poor health** | **Intermediate health** | **Ideal health^a^** |
| **Current smoking** | Yes | Quit <2 years | Never or quit ≥2 years^d^ |
| **BMI** | ≥30 kg/m^2^ | 25-29.9 kg/m^2^ | <25 kg/m^2^ |
| **Physical activity** | None | 1-149 minutes moderate  or 1-74 minutes  vigorous or 1-149  minutes moderate+ vigorous per week | ≥150 minutes moderate or ≥75 minutes vigorous or ≥150 minutes moderate+  vigorous per week |
|  |  |  |  |
| **Total cholesterol** | ≥6.2 mmol/L^c^ | 5.2-6.1 mmol/L^c^ | <5.2 mmol/L^c^ |
|  |  |  |  |
| **Blood pressure** | SBP ≥140 mmHg^c^ | 120 ≤ SBP <140 mmHg or (SBP <120 and  DBP ≥80 mmHg)^c^ | SBP <120 &  DBP <80 mmHg^c^ |
| **Fasting blood glucose** | Have diabetes | N/A | No diabetes |
|  |  |  |  |
|  |  |  |  |
| **Score of CHM** | 0 | 1 | 2 |
|  |  |  |  |
| **Presence of ICH** | No | No | Yes |

^a^ The definition of ideal health of each CHM is the definition of ICH.

^b^ Treatments included medications for CVD and medications for adverse health factors, such as, high blood pressure and high blood cholesterol.

^c^ As values of blood pressure and total cholesterol were corrected for medication use, no further adjustment was made using the AHA standard conventions for such metrics.

BMI: body mass index. CHM: Cardiovascular Health Metrics. DBP: diastolic blood pressure. ICH: Ideal Cardiovascular Health. N/A: not available. SBP: systolic blood pressure.
Adapted and modified from Lloyd-Jones et al. [7]

^d^ Never or quit ≥2 years implies non-smokers and those who were abstinent from smoking for the last 2 years.

**Supplement Table 4. Selected characteristics of all 4330 participants and the subset with frailty**

| **Mean (SD) or n (%), unless specified** | | | **All participants** | **Frailty cases** |
| --- | --- | --- | --- | --- |
|  | | |  |  |
| Demography/medical history | | |  |  |
|  |  | |  |  |
|  | No. of participants | | 4330 | 407 |
|  | Age, yrs, median (IQR) | | 60 (55-67) | 67 (60-75) |
|  | Male | | 1928 (44.5) | 126 (31.0) |
|  | Current smoker | | 674 (15.6) | 82 (20.2) |
|  | Physical activity, minutes/week, median (IQR) | | 150 (0-720) | 0 (0-210) |
|  | Diabetes | | 246 (5.7) | 68 (16.7) |
|  | BP-lowering medication | | 1229 (28.4) | 251 (61.7) |
|  | Lipid-lowering medication | | 942 (21.8) | 165 (40.5) |
|  | SBP, mmHg | | 138.3 (20.9) | 144.7 (21.7) |
|  | DBP, mmHg | | 84.4 (11.4) | 85.2 (11.7) |
|  | Total cholesterol, mmol/L | | 5.5 (1.0) | 5.4 (1.1) |
|  | BMI, kg/m^2^, median (IQR) | | 28.0 (25.2-31.2) | 29.9 (26.9-33.9) |
|  |  | |  |  |
| Cardiovascular risk scores | | |  |  |
|  |  | |  |  |
|  | SCORE, % | |  |  |
|  |  | Low-to-moderate (<5) | 3153 (72.8) | 211 (51.8) |
|  |  | High risk (5-<10) | 710 (16.4) | 102 (25.1) |
|  |  | Very high risk (>10) | 467 (10.8) | 94 (23.1) |
|  | ICH (Max. 6) | |  |  |
|  |  | Inadequate (0-2) | 1308 (30.2) | 201 (49.4) |
|  |  | Average (3) | 1653 (38.2) | 146 (35.9) |
|  |  | Optimal (4-6) | 1369 (31.6) | 60 (14.7) |
|  | CHM (Max. 12) | |  |  |
|  |  | Inadequate (0-5) | 626 (14.5) | 146 (35.9) |
|  |  | Average (6-7) | 1520 (35.1) | 150 (36.9) |
|  |  | Optimal (8-12) | 2184 (50.4) | 111 (27.3) |

BP: blood pressure. CHM: Cardiovascular Health Metrics. DBP: diastolic blood pressure. ICH: Ideal Cardiovascular Health. IQR: interquartile range. SBP: systolic blood pressure. SCORE: Systematic Coronary Risk Evaluation. SD: standard deviation.

**Supplement Figure 1. CONSORT diagram of all participants and subset with no prior history of CVD**

Total participants

n=8504 at baseline

Available sample

n=5888

Eligible sample

n=4330

Analysed sample

n=5618

Excluded (n=270):

Missing data on outcomes

Excluded (n=1288):

1. Parkinson’s diseases (n=26)
2. MMSE<18 (n=13)
3. Stroke (n=84)
4. Use of antidepressants (n=348)
5. A history of prior CVD other than stroke (n=817)

Excluded (n=2616):

1. Did not attend a health assessment (n= 2350)
2. Age <50 years (n= 256) or missing data on age (n=10)

MMSE: Mini-Mental State Examination.

**Supplement Figure 2. Quantile-quantile plot of the frailty index in 4330 participants versus Gamma distribution with scale parameter *λ* (16.26) and shape parameter *k* (2.02)**

**
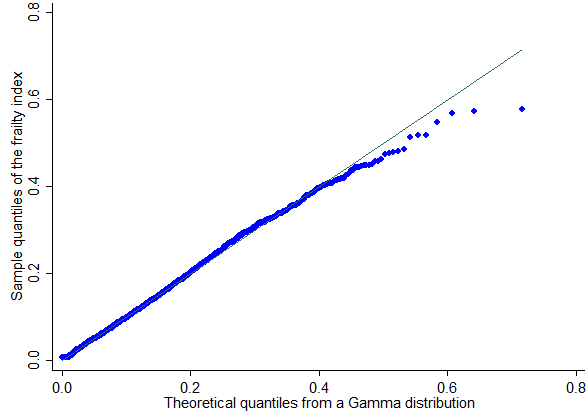
**

**Supplement Figure 3a. Frequency distribution of 40-item components of the frailty index in 4330 participants with no prior history of CVD**


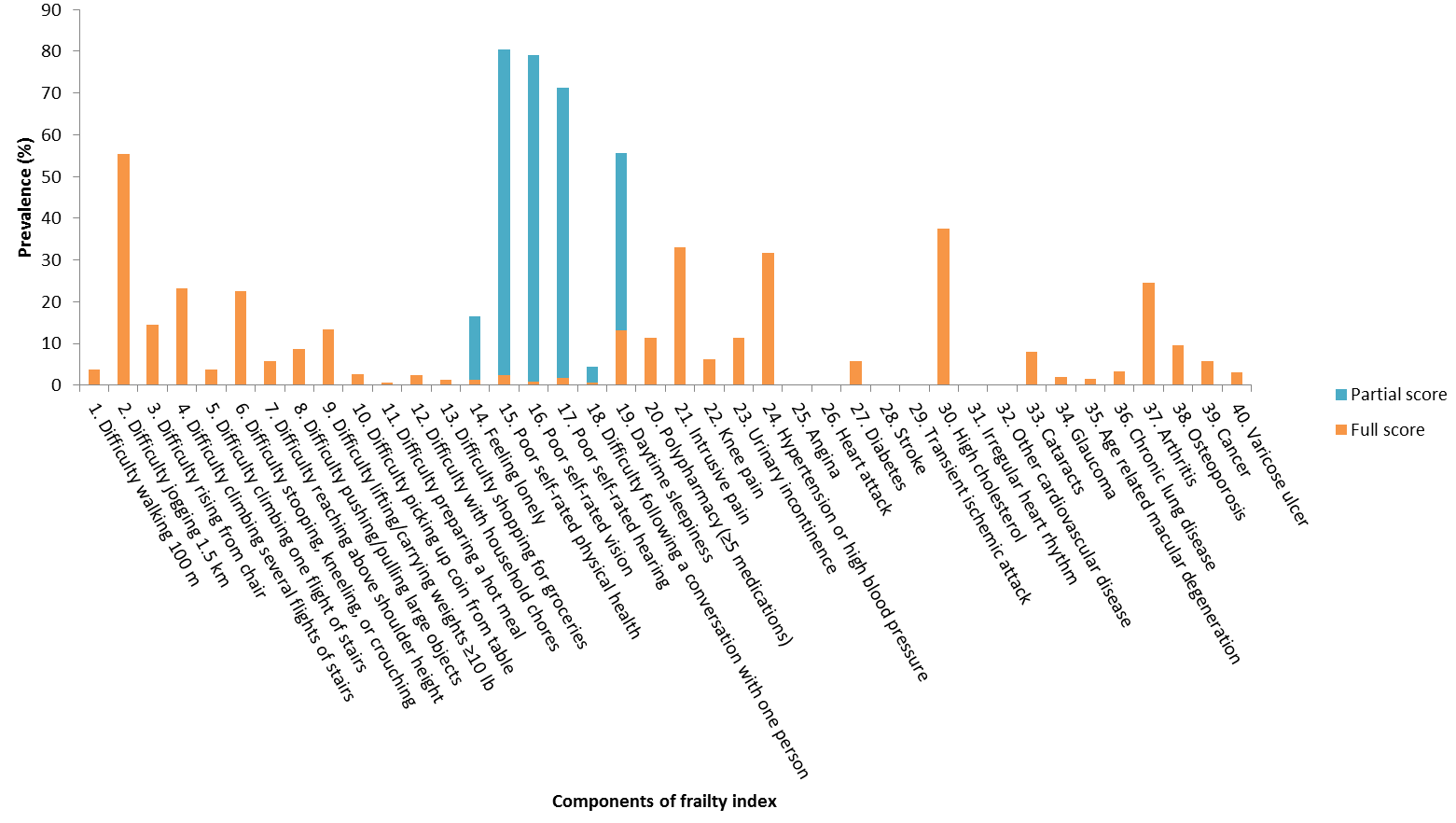


For categorical deficits, the proportions of full (i.e. one component) and partial deficits (between 0 and 1 component) are presented.

**Supplement Figure 3b. Frequency distribution of 40-item components of the frailty index in all 5618 participants**


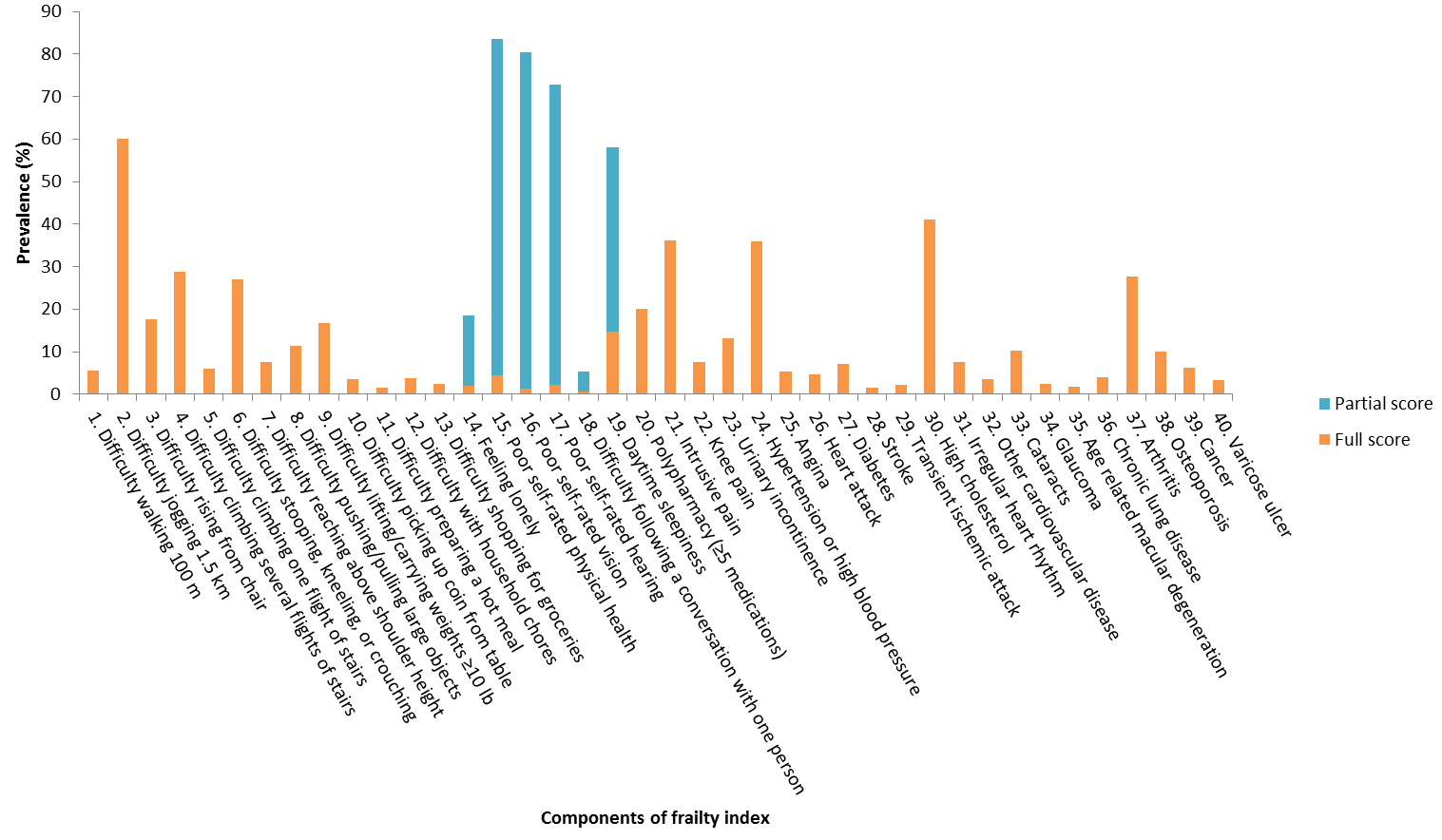
For categorical deficits, the proportions of full (i.e. one component) and partial deficits (between 0 and 1 component) are presented.

**Supplement Figure 4. Association of systemic coronary risk evaluation with frailty, without correction for use of medication**

Odds ratios (OR) are presented on a floating absolute scale. Each square has area inversely proportional to the variance of the log OR. The horizontal lines indicate 95% confidence intervals. The vertical line corresponds to an OR of 1.0.

**Supplement Figure 5. Associations of Ideal Cardiovascular Health (ICH) and Cardiovascular Health Metrics (CHM) with frailty, without correction for medication**

Symbols and conventions as in Supplement Figure 4.
